# From hidden springs to endless oceans: exploring the complementary roles of the amygdala and hippocampus in phenomenal experience

**DOI:** 10.1093/nc/niag007

**Published:** 2026-03-12

**Authors:** Ronald Sladky

**Affiliations:** Social, Cognitive and Affective Neuroscience Unit, Department of Cognition, Emotion, and Methods in Psychology, Faculty of Psychology, University of Vienna, Liebiggasse 5, 1010 Vienna, Austria

**Keywords:** self-consciousness, minimal phenomenal experience, non-dual awareness, ego death, neurofeedback, fMRI, amygdala, hippocampus, neuroanatomy, neuroimaging

## Abstract

Current theories of consciousness often emphasize its ego-centric functions, highlighting the role of the insular cortex in interoceptive self-modeling and subcortical brain regions in qualitative experience and motivation, aptly described as the ‘hidden spring’ of consciousness. From ecological and pragmatic perspectives, conscious experience may facilitate the self-organization of complex organisms by optimizing goals that are typically parallel, multifaceted, and difficult to reconcile. However, the notion that all forms of conscious experience are ego-centric, or at least grounded in a minimal sense of self, is challenged by credible reports of minimal phenomenal experience (MPE), which occur without any self-referential content. I propose that this apparent duality in conscious experience can be explained by the dual-origin theory of cortical development. This theory suggests a gradual expansion of cortical cytoarchitecture from two distinct subcortical origins. The ‘Amygdala-System’ supports interoceptive self-modeling for habitual interactions with the body and the environment. It expands ventrally from the olfactory system and amygdala, enabling ego-centric processing. In contrast, the ‘Hippocampus-System’, centered on the hippocampus and expanding dorsally, supports allocentric cognition and experiences that are not constrained by self-referential processing. This complementary system allows for open-ended, selfless forms of experience, akin to an ‘endless ocean’. In this framework, MPE may represent a fragile form of consciousness, typically overshadowed by the self-related interoceptive and exteroceptive functions of the Amygdala-System. Finally, I discuss how real-time functional magnetic resonance imaging (fMRI) neurofeedback could be used to upregulate the Hippocampus-System, potentially enabling the controlled study of MPE in neuroscientific settings.

**Highlights**

- Evidence from developmental neuroanatomy suggests two different subcortical origins of the mammalian cerebral cortex, potentially entailing two modes of conscious experience.
- An amygdala-centered developmental gradient could allow for ego-centric functions relevant for adaptive self-regulation including survival in a physical and social world.
- A hippocampus-centered system could allow for allo-centric world modeling, which is less constrained by an ego-centric perspective.
- Several hypotheses are proposed to investigate the neuroanatomical and phenomenological claims by using real-time functional MRI-based neurofeedback in a controlled experimental setting.

## Introduction

In his book, *The Hidden Spring*, Mark Solms made several noteworthy claims about conscious experience that are in line with several ecological and neuroscientific desiderata: “Feelings are real, and we know about them because they permeate our consciousness. […] From their origin in some of the most ancient strata of the brain, they irrigate the dead soil of unconscious representations and bring them to mental life.” (Solms 2021). Similarly, Cleeremans and Tallon-Baudry argue that only because “conscious agents experience things and care about those experiences” they are “motivated to act in certain ways” and “prefer some states of affairs vs. others” (Cleeremans and Tallon-Baudry 2024). Anil Seth’s explanation of the origin of consciousness also reflects this care for the self, proposing that consciousness arises from embodied, control-oriented predictive regulation, where “experiences of having a body, and of simply ‘being’ a body, along with the many and varied elements of selfhood, are Bayesian best guesses designed by evolution to keep us alive” (Seth 2021). Phenomenal experience arises from feelings that carry intrinsic meaning for an organism, expressed through positive or negative valence, making such experiences both subjective and actionable. Solms further emphasizes that complex organisms benefit from qualitative conscious states, as these enable them to model intricate processes that go beyond basic quantitative regulatory mechanisms, such as reflex arcs or homeostatic regulation. I regard this self-serving perspective on phenomenal experience as a compelling ecological argument, as it provides agents with intrinsic value to guide optimal decision-making. This, in turn, offers evolutionary fitness advantages for complex multicellular organisms navigating challenging eco-niches and solving nontrivial optimization problems in precarious situations far from thermodynamic equilibrium. Consistent with this theory, rooted in precarious and predictive self-regulation, Solms proposes that the origin of consciousness lies not in cortical processes but in subcortical regions, such as the brainstem and its connected areas. Supported by numerous lesion studies in clinical cases, he argues that these subcortical regions serve as the ‘hidden spring’ of motivational drive and conscious experience (Solms 2021).

If the core of all phenomenal experience is the phenomenal self, the most minimal phenomenal experience (MPE) should be centered around a ‘Me’, the experience of having an objective body, or an ‘I’, the experience of being a subjective experiencer. William James, however, already pointed toward the option that MPE might be what he called ‘Sciousness’, a form of content-less “instant field of the present,” free of any subject/object duality. This claim has been recently backed up by credible reports of experiential states without any subjective self or agency (Metzinger 2024) described as pure awareness, the simplest form of conscious experience, i.e. “an experience of awareness itself that is not even subjective in the sense of being tied to a consciously experienced first-person perspective or a personal-level self-model anymore.” (Gamma and Metzinger 2021). Could there be phenomenal experience without a subject, value, and agency? If so, how would it arise in a nervous system that appears to be driven by self-regulation and maintaining its perspectival reality?

I argue that the strong evidence for self-less experiences during MPE does not necessarily negate the possibility that at least parts of the nervous system are centered on self-regulation and self-modeling, with this function predominantly shaping conventional phenomenal experience. However, MPE may be facilitated by brain networks that become functionally decoupled from self-centered interoceptive processes in regions such as the brainstem, insula, or amygdala. Based on the dual origin hypothesis for cortical development and evolution (Sanides 1962), I propose the hippocampus and connected brain areas as a candidate to allow for these allocentric experiences to arise. The Rethinking Cortical Organization section will review perspectives on brain organization guided by the principles of cortical development. The Amygdala-System for Egocentric Self-Modeling section will introduce a system of cortical areas that have their origin in an amygdala-centric expansion gradient (Amygdala-System), which are consistent with ego-centric brain functions, such as affectivity, interoception, and subjective value. The Hippocampus-System and Allocentric Modeling section will discuss a hippocampus-centric expansion gradient (Hippocampus-System) that allows for allocentric world-modeling. The Amygdala- and Hippocampus-System Interactions section will discuss how the Amygdala- and Hippocampus-Systems typically interact during non-MPE, while the Methods and Empirical Predictions section will discuss empirical predictions for their interaction during MPE and how they could be introduced experimentally.

## Rethinking cortical organization

Solms and others have argued that it is time to reconsider the historical overemphasis on the importance of cortical structures in understanding brain functions, such as consciousness. For instance, the traditional view of visual perception is often described as a process of abstraction from raw sensory input: photons trigger neural responses in the retina, which activate the optic nerve and relay signals through the lateral geniculate nucleus to the visual cortex for feature detection. From there, the information is thought to progress to association cortices, where semantic concepts are formed. This proposed neural hierarchy suggests a linear progression from simple to complex, from noisy sensory fluctuations to stable regularities, and from primitive automatic responses to sophisticated, deliberate thought. While this framework has been highly influential, offering a functional roadmap for cognitive neuroscience and engineering, this stimulus-driven view largely ignores how top–down predictions can shape perceptions. Predictive coding theories (Rao and Ballard 1999) challenge the traditional feed-forward hierarchy by emphasizing the role of top–down processes. Expectations and prior knowledge generate predictions that guide perception in an energy- and data-efficient manner. For example, because we live in a social world, identifying people around us is crucial for survival. This predisposes our perception to prioritize detecting faces, even when they are partially occluded or ambiguous, though this comes at the cost of occasionally misclassifying random patterns as faces. While predictive coding offers a compelling framework for understanding the salience of important stimuli and their efficient encoding within a generative, hierarchically distributed model, an overly cortex-centered perspective seems to overlook the underlying drivers and mechanisms that shape this process. Cortical functions are often described as being influenced by factors such as emotions, motivation, and attention. However, could it be that these very factors are not merely modulators but the primary drivers of cortical processes themselves?

Subcortical cerebral regions, which are central to emotions, motivation, and attention, the hallmark functions of self-relevant cognition, are phylogenetically older than the neocortex and characterized by simpler cytoarchitecture. In contrast, the prefrontal cortex supports functions traditionally associated with “higher” cognition, such as language production, based on the assumption that these abilities are hallmarks of “higher” species like humans. However, there are important functional and anatomical reasons to challenge this anthropocentric narrative. From a biological standpoint, these so-called “higher” functions often have limited relevance for an organism’s survival within its ecological niche (Brooks 1990). This calls for a reevaluation of how such labels contribute to progress in neuroscience (Pessoa *et al*. 2022). In fact, there is mounting evidence that subcortical brain areas play an important role in regulating resting state brain connectivity (Li *et al*. 2021) and they are involved in essentially all higher cognitive functions (Janacsek *et al*. 2022). Functional and neuroanatomical evidence has led to reconceptualized models that place the so-called limbic system on top of the computational hierarchy, given its relevance for maintaining allostatic control (Chanes and Barrett 2016; Tucker and Luu 2021; Katsumi *et al*. 2022; Luu *et al*. 2024). In contrast, ontogenetically and phylogenetically younger cortical areas appear to enhance computational and storage capacity, providing greater behavioral flexibility. Notably, cortical lesions often preserve stereotypic, survival-essential behaviors while impairing more complex, yet less critical, brain functions.

Building on the seminal work of Brodmann and Talairach/Tournoux, who differentiated cortical areas based on anatomical microstructures, human brain mapping has traditionally focused on identifying distinct brain regions, resulting in a mosaic of localized brain functions. However, an alternative approach, first pioneered by Vogt and Vogt-Mugnier in the nineteenth century, emphasizes differences in cortical types based on variations in laminar structure. This perspective holds significant promise as a complementary framework for understanding brain function, particularly in the context of brain evolution and maturation (Sebenius *et al*. 2025). Increasingly, neuroimaging research is turning its attention to the trajectories of ontogenetic and phylogenetic brain development through the lens of functional gradients (Bernhardt *et al*. 2022), leading to a renaissance of some older theoretical proposals on brain organization and function.

**Figure 1:**
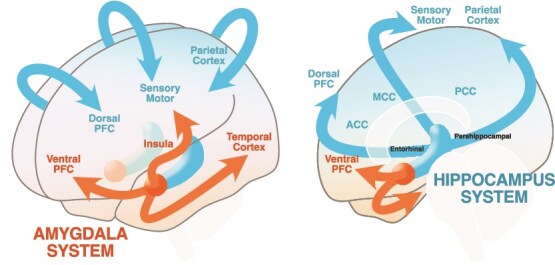
Schematic illustration of the dual origin hypothesis. The Amygdala-System represents the amygdala developmental expansion gradient toward the ventral cerebral cortices, e.g. the insula cortex (agranular, dysgranular) and operculum to the ventral sensorimotor cortex (eulaminate and koniocortical). The Hippocampus-System represents the hippocampal gradient *via* cingulate and parahippocampal cortices (agranular, dysgranular) toward the dorsal cerebral cortices (eulaminate and koniocortical).

One of these cortical-type perspectives is Sanides’ dual origin hypothesis of cortical development (Sanides 1962; see Chanes and García-Cabezas 2025 for contextualization), which suggests that younger, more differentiated cortical areas gradually emerged from two different expansion points. One system originated from the olfactory system, resembling an amygdala-centered expansion gradient toward ventral cortical areas, which could enable interoceptive self-modeling for allostatic control and habitual interactions with the body and the environment (Amygdala-System, Fig. 1). The other system is hippocampus-centered, expanded toward more dorsal cortical areas, and could enable allocentric forms of cognition and experience (Hippocampus-System, Fig. 1).

Both systems share a common feature: an increase in neuroanatomical complexity along their expansion gradients. This progression reflects a transition from older structures with fewer neural layers to newer structures with more differentiated layers. At the starting point of the gradient lie the primordial limbic zones, referred to as the allocortex. The allocortex can be separated into an ancestral hippocampal sector and the olfactory sector, which I refer to as the origin of the Amygdala-System. Contemporary genoarchitectonic studies, which use *in situ* hybridization to label the expression of genes in the developing cortex of mouse embryos, have shown that allo-, meso-, and isocortical sectors are specified in concentric rings (Puelles *et al*. 2019), as originally proposed by Sanides, and have specific genetic profiles in the adult mouse cortex (Puelles *et al*. 2024). Two ‘cortical organizers’ (i.e. cells that release morphogenetic proteins to the adjacent extracellular medium to induce a particular kind of neuronal growth and organization gradient) have been identified that correspond to the ancestral hippocampal sector (expression of the morphogenetic genes Left1 and Lhx2) and the olfactory sector (expression of Tbr1) (Subramanian *et al*. 2009).

Going outside along the direction of this neurodevelopmental gradient, the primordial limbic allocortical areas are followed by the three-layered paralimbic areas, such as the piriform, entorhinal, parahippocampal, and ventral cingulate cortices. Further along the gradient are regions with either no granular layer IV (e.g. agranular insular and precentral cortices) or only a rudimentary granular layer IV (e.g. dysgranular insular, dorsal cingulate, and orbitofrontal cortices). At the apex of this gradient is the six-layered cortex, known as the isocortex or neocortex, which is unique to primates and exhibits the highest degree of laminar differentiation (Giaccio 2006; García-Cabezas *et al*. 2022), causally explainable by the action of the two cortical organizers.

Functionally, I propose that the two principal expansion points, amygdala (originating in the ancestral olfactory system) and hippocampus, serve as the top level of a cortical and functional hierarchy, encapsulating the core functions of their network in the most abstract form. The other regions along the developmental gradient implement increasingly finer-grained details, enabling greater behavioral and perceptual flexibility. These two distinct developmental origins may give rise to complementary, functionally specialized systems that are nonetheless integrated at multiple levels. I will review the potential functions of the Amygdala- and Hippocampus-Systems, as well as how they interact.

## Amygdala-System for egocentric self-modeling

Initially, amygdala research focused mostly on its relevance for threat, fear, and anxiety, typically in Pavlovian settings. In this regard, we have learned that the amygdala not only prepares downstream physiology for Pavlovian responding but also contributes to the subjective experience of emotions and highly complex social communication (LeDoux 2003; Tranel *et al*. 2006; Adolphs 2010). Later, the amygdala has been appreciated for its role in negotiating both appetitive and risky behaviors and motivations and thus plays a role for both positive and negative affect and emotions. In this regard, the amygdala emerged as an integrated network component that encodes emotions (Amaral and Adolphs 2016; Inman *et al*. 2020). The amygdala itself consists of several smaller subnuclei (Janak and Tye 2015) that exhibit marked differences in connectivity and function, which themselves consist of several cell types wiring up complex interconnected neuronal networks within and across these structures with complex dynamics and multiple functions. Adding to that, within a subnucleus, cell populations with varying cell types, gene expression, and functions inhabit the same small space (Babaev *et al*. 2018; Hur *et al*. 2020). Finally, there is good reason to include the distal bed nucleus of the stria terminalis into a macrostructure termed extended (central) amygdala (Alheid and Heimer 1988; Fox *et al*. 2015), as both can encode and control both negative and positive affective states and behaviors, even though it is not inside the compact almond-shaped amygdaloid structure and separated by the internal capsule. On an anatomical level, the internal and external boundaries appear gradual and pending on the level of analyses (e.g. cellular makeup and connectivity). On a functional level, these differentiations between the subnuclei and their microcircuitry become apparent. This was demonstrated by rodent research and even with noninvasive human functional magnetic resonance imaging (fMRI) studies, e.g. between the basolateral and central amygdala (Sladky *et al*. 2021b) or the central amygdala and the bed nucleus (Torrisi *et al*. 2018).

The updated view is that the amygdala functions as a central hub for multimodal and multisensory integration, positioning it near the top of a self-relevant hierarchical active inference model. This framework helps explain why amygdala activation, such as prediction errors arising from deviations from homeostatic preference priors, can lead to profound changes in both behavior (e.g. avoidance or approach behaviors) and subjective experience (e.g. fear and other emotions). In a recent review, I explored the amygdala’s role in homeostatic and allostatic control, as well as its role in perception, from an active inference perspective (Sladky *et al*. 2024), and how it is ideally positioned for linking interoceptive regulation (*via* inputs from brainstem and interoceptive models in the insular cortex) with exteroception (*via* temporal lobe and thalamus). Amygdala output, in turn, plays a critical role in modulating behavior through multiple pathways. These include the brainstem (e.g. rapid, stereotypical threat responses and regulation of the autonomic nervous system), the ventral striatum (e.g. biasing action selection), the prefrontal cortex (e.g. enabling active avoidance that requires long-term planning), and the hypothalamus (e.g. inducing somatic arousal *via* hormonal cascades).

The central extended amygdala, which includes the bed nucleus of the stria terminalis, has its origin in the subpallium, a subdivision of the embryonic telencephalon, which also comprises the striatum and parts of the olfactory system. Subpallial structures can already form a simple system that mediates approach and avoidance behavior and valenced perception: for example, pleasant smells, such as those indicating the presence of food or social partners, can elicit approach behaviors. In contrast, aversive or pungent odors may signal danger, such as the proximity of predators or rivals, leading to avoidance behaviors, negative affective states, and heightened alertness. The effects of olfactory input on the medial amygdala are well studied in rodents, highlighting its fundamental role in survival (Wernecke *et al*. 2015). However, even though the sensory input of breathing plays a role in modulating intrinsic amygdala activity (Boyadzhieva and Kayhan 2021), vision and other senses appear to play a dominant role in human amygdala functions. Waymel et al. argue that the stable and reliable access to food sources has driven a volume and connectivity reduction within the olfaction system during brain evolution and development (Waymel *et al*. 2020), while certain amygdala subnuclei such as the basolateral amygdala underwent a massive phylogenetic expansion (Janak and Tye 2015). This suggests that the amygdala, originally an organ for valenced olfaction, has retained its importance but shifted more toward multimodal and social functions. Consequently, even though rodent genoarchitectonic studies highlight the olfactory system as one of the cortical organizers, at least in primates, it is appropriate to functionally refer to it as the Amygdala-System.

While the central amygdala is subpallial in origin, the basolateral part of the amygdala develops from the pallial subdivision of the embryonic telencephalon (like the hippocampus). The phylogenetic expansion of the basolateral amygdala in primates (Janak and Tye 2015) allows for models with higher hierarchical width, leading to more fine-grained, multimodal perception and increased action flexibility. The differences in anatomical microstructure parallel functional (sometimes complementary) specializations that are often overlooked if not accounted for, as we previously demonstrated for the amygdala in social cognition (Sladky *et al*. 2021b). Functionally defined subparcellations could delineate the complex cytoarchitecture of subcortical brain areas, which underwent a long evolutionary history of model optimization (Garcia-Calero and Puelles 2020).

The amygdala gradient, originating near the basolateral amygdala, extends in three main directions:

1. *From the amygdala via the insula cortex and operculum to the sensorimotor cortex.* This gradient would be a good candidate for interoceptive functions and self-regulations. The expansion gradient terminates in the ventral part of sensorimotor cortex, which ia relevant for face movements and sensations, important parts of affective behavior and experience.
2. *From the amygdala to the posterior temporal lobe.* This gradient reflects the ventral perceptual stream. The so-called *What?*-pathway in vision is for identification of visual stimuli from simple concrete (posterior part) toward more complex features (anterior part) and relevance detection (amygdala). Importantly, it exhibits regions that are optimized for specialized feature detection (e.g. faces). This expansion gradient plays and important role in exteroception. We assume the basolateral amygdala plays a role in tuning hierarchical feature selection (Sladky *et al*. 2024). A similar gradient exists for auditory processing.
3. *From the amygdala to the ventral prefrontal cortex.* The ventral PFC is known to play a role in what is traditionally called prefrontal-amygdala regulation in affective neuroscience (Sladky *et al*. 2015a, 2021a). However, while most of the orbitofrontal cortex is Amygdala-System, the most medial part, the gyrus rectus, belongs to the Hippocampus-System (García-Cabezas and Barbas 2016; Chanes and García-Cabezas 2025).

The central amygdala is frequently regarded as the primary cause for the initiation of motivated behavior. The expression of stereotypical defensive behavior *via* the autonomic nervous system, hormonal cascades, and brainstem mechanisms (Freese and Amaral 2009; Kong and Zweifel 2021) is reduced by amygdala lesions and intake of benzodiazepines (Griessner *et al*. 2021).

Lesions in the (basolateral) amygdala, e.g. due to Urbach–Wiethe disease (Koen *et al*. 2016), leads to hypervigilance, including a lower threshold for fearful face recognition and longer gaze duration to the eye region toward fearful face stimuli (Terburg *et al*. 2012). Relevant for self-centered processes, people affected report almost no fearful emotions (Amaral and Adolphs 2016) and more positive autobiographic memories (van Honk, personal communication) and dream experiences (Blake *et al*. 2019). They are described as exceptionally prosocial, without much ego-centric concern, in general, leading to more generous economic investments in trust games (van Honk *et al*. 2013) but reduced ability to identify and sanction harmful people (Rosenberger *et al*. 2019) and inflexible, nonutilitarian judgments even in the most extreme cases (van Honk *et al*. 2022). In neurotypical people, in turn, nonaggressive and prosocial behavior is mediated by the ventromedial prefrontal cortex (Beyer *et al*. 2015; Lockwood *et al*. 2024), which is a part of the Hippocampus-System and involved in prefrontal amygdala down-regulation. Downstream lesions along the Amygdala-System gradient do not result in a complete loss of function but rather in the development of impoverished, less flexible models. For example, medial prefrontal cortex lesions lead to impairment in emotion regulation (Bechara *et al*. 1999). Insular lesion impairs interoception but does not prevent bodily experiences (Solms 2021)^1^.

## Hippocampus-System and allocentric modeling

Like the amygdala, the hippocampus is part of a subcortical brain system with evolutionary, highly preserved microcircuits. However, over the course of primate brain evolution and simultaneous gain of function by specialized cortical connectivity, the integration of the hippocampus within the rest of the brain has undergone fundamental reconfiguration (Eichert *et al*. 2024). As the second part of the pallial allocortex, the hippocampus expands radially in several directions toward the dorsal neocortical area, e.g.:

1. *From hippocampus to anterior cingulate cortex and dorsal prefrontal areas (e.g. dorsolateral prefrontal cortex).* Functionally, this gradient plays an important role in the formation of declarative, associative memories, and relating new to old information (Menon 2016). After acquisition, as soon as memories are established in cortical areas, the functional relevance of the hippocampus is decreased (Eichenbaum 2017) and, eventually, no longer required during the retrieval processes (Qin *et al*. 2014).
2. *From the hippocampus to the parahippocampal cortex and parietal lobe.* Historically, the hippocampus has been studied extensively in the context of navigation. Place cells in the hippocampus (and grid cells in the adjacent entorhinal cortex) dynamically encode the current location. During memory consolidation, the parietal cortex encoding changes to a more permanent and action-oriented format (Whitlock *et al*. 2008). Following an abstraction gradient, the hippocampus encodes abstract coordinate information, while the parietal cortex is crucial for more concrete, categorical spatial relationships (Baumann and Mattingley 2014).
3. *From the hippocampus to the medial cingulate cortex and the dorsal sensorimotor network.* The sensorimotor network is a mostly cortical network that includes primary and sensory-motor areas, as well as supplementary motor areas (Yeo *et al*. 2011). As such, the world model from the hippocampus network enables coordinated, goal-directed motor actions.

A model of space and time, derived from real-life experiences, appears to be the basis for encoding of episodic memories. Hippocampus encodes spatial and nonspatial relationships not as a static mapping but in the form of relational constructions, neuronally implemented by dynamic re-entrant loops and amorphous networks (Maurer and Nadel 2021). The hippocampus is involved in sequence learning, which can be seen as the basis for an active construction of temporal order and duration (Bellmund *et al*. 2022), while interoceptive expectations and physiological signals inform temporal modeling in the Amygdala-System. Considering that the Hippocampus-System provides a world model with several layers of abstraction, its involvement in mathematical cognition is unsurprising. Prefrontal areas appear to be involved in rule-based and context-specific manipulations of quantity-related information encoded in the parietal cortex, whereas activation in temporal regions relates to reading/hearing mathematical symbols (Menon 2016).

Functional brain connectivity shows that different subfields of the hippocampus connect to different areas of the cortex, not only to the sensorimotor resting-state network but also to the default mode network (DMN; Ezama *et al*. 2021), heteromodal regions in the neocortex, including Hippocampus-System areas such as the dorsolateral prefrontal and parietal cortices, involved in mind wandering and sensory monitoring. Furthermore, the DMN appears to be involved in some form of self-modeling (Davey *et al*. 2016), including autobiographical memory, future thinking, and self-perception (Eichert *et al*. 2024). Changes in self-experience also affect DMN connectivity (Smigielski *et al*. 2019). I argue that this form of self-modeling is primarily centered around a narrative self, how I perceive myself in relation to the external world, what William James referred to as the *Me*. In contrast, Amygdala-System’s ego-centric model, the *I*, is rooted in interoceptive sensations and the self-model. The involvement of distinct brain networks in these two types of self-processing is reviewed in detail by Qin *et al*. (Qin *et al*. 2020); in the following section, I will explain interactions between Amygdala- and Hippocampus-Systems on a more principled level.

## Amygdala- and Hippocampus-System interactions

A central argument of my proposal is that there are two independent functional systems arising from an amygdala- and a hippocampus-centered brain network. For example, in visual perception, they perform complementary functions: the Amygdala-System corresponds to the ventral *What?*-pathway for identifying relevant objects and the Hippocampus-System to the dorsal *Where?*-pathway for localizing and tracking objects (Mishkin and Ungerleider 1982). According to the proposed architecture, the highest hierarchical level of the *What?*-pathway is the amygdala and responsible for self-relevant top–down predictions to increase the detectability and salience of ecologically relevant sensory features (e.g. indicating subjective value, novelty or threats) and to initiate behavior that leads to desired outcome (e.g. obtain rewards, avoid threats) (Sladky *et al*. 2024). The expansion outwards on the ventral *What?*-pathway resembles a gradient toward more detailed and specific but degenerated and ambiguous sensory features with less inherent self-focus. The dorsal *Where?*-pathway appears to be reversed when it comes to self-relevance. The dorsal stream in the parietal cortex does not directly encode abstract spatial information but more self-centered concrete models of motor control (Goodale and Milner 1992; Tucker and Luu 2012) to enable different forms of object grasping (Luppino and Rizzolatti 2000; Rizzolatti and Matelli 2003; Luu *et al*. 2024). Abstraction, i.e. placing these sensory-motor models into an abstract allocentric spatial model, happens along the gradient toward the center of the hierarchy. It is noteworthy that this world modeling may not be entirely nonperspectival, as head direction cells are well established to influence spatial coding in the hippocampus (Taube 1998). While they may introduce physical egocentricity, which enhances the efficiency of encoding space and action, their involvement does not necessarily entail egocentric self-identification with that perspective. Finally, the hippocampus, known for its role in encoding spatial information, establishing relationships between objects, and creating maps, is the site where the highest level of abstraction or generalization occurs.

While I have reported on evidence for their complementary functions, I also need to stress that these networks are most likely never truly dissociated during everyday cognition. Along the neuroanatomical expansion gradient, reciprocal interactions between Amygdala- and Hippocampus-Systems happen on several levels between sectors of equivalent architectonic development. Already on the most basic level, the hippocampus connects to the olfactory system and, in fact, the link between smells and memory reactivation is well known (Waymel *et al*. 2020). More particularly, the connection between the olfactory piriform cortex and hippocampus is thought to be relevant for odor–place associations and olfaction-guided spatial navigation (Poo *et al*. 2022). Similarly, despite the preference for the Amygdala-System, the primate amygdala is reciprocally connected with mesocortical areas of both gradients (Barbas and De Olmos 1990; Ghashghaei and Barbas 2002; Ghashghaei *et al*. 2007). Amygdalar connections are generally excitatory and more extensive than the hippocampus, which appears more important for inhibition to limit the organism’s stress response using mechanisms based on contextual episodic and spatial memory, e.g. contextual conditioning, habituation, and extinction (Giaccio 2006). A hippocampal subfield resting-state fMRI study suggests the existence of a connection between the hippocampal sensorimotor cortex network and the amygdala (and orbitofrontal cortex) (Ezama *et al*. 2021). As shown using intracranial recordings, synchronization between the amygdala and the hippocampus and connected cortical networks fluctuates across different sleep phases (Muñoz-Torres *et al*. 2023). Motivated perception, e.g. how intrinsic motivation for salient stimuli drives spatial attention orientation in the hippocampal–parietal network, illustrates how the amygdala influences the Hippocampus-System. In general, lesions in the core of the Amygdala-System disturb the motivation of behavior, while core Hippocampus-System lesions disturb the temporospatial organization of action (Giaccio 2006). Basolateral amygdala lesions do not impair spatial cognition but affect attention during face perception, leading to both reduced salience, failure to initially orient attention toward the stimulus (Jha *et al*. 2023), and subsequent hyperfocus, i.e. failure to re-orient attention away from the face stimulus (Terburg *et al*. 2012). Neuronally, there is hyperactivation in the parietal cortex and other Hippocampus-System areas (Hortensius *et al*. 2016) such as the subgenual anterior cingulate and dorsomedial prefrontal cortices but deactivation of Amygdala-System areas such as the temporal cortex (Hortensius *et al*. 2017).

While there is a strong functional relationship between the amygdala and insula (Amygdala-System), there is no strong evidence for connectivity of different subparts of the insula (dysgranular/agranular) and hippocampus (allocortical), entorhinal cortex (paralimbic), and other core Hippocampus-System areas (Gehrlach *et al*. 2020). Instead, the insula interacts with more differentiated areas along the Hippocampus-System gradient, with similar anatomical structure, such as the anterior cingulate cortex (Seth and Friston 2016).

In the prefrontal cortex, there are Amygdala-/Hippocampus-System interactions between ventromedial and dorsomedial cortices, ventrolateral and dorsolateral prefrontal cortices. Functionally, it has been suggested that a reciprocal equilibrium between these prefrontal sectors plays a central role in the regulation of mood, both in neurotypical and clinical populations (Giaccio 2006). For example, during emotional faces discrimination, ventral PFC (Amygdala-System) exhibits stronger amygdala regulation than dorsal PFC (Hippocampus-System) (Sladky *et al*. 2021a) but is lower in social anxiety disorders (Sladky *et al*. 2015a) yet higher after SSRI intake (Sladky *et al*. 2015b). Reappraisal, which can be seen as an egocentric self-regulation strategy, is facilitated by ventrolateral PFC, while dorsolateral PFC is involved in distraction strategies, i.e. directing attention away from the present egocentric experience (Zhao *et al*. 2021). Pertaining to Hippocampus-System function, the ventrolateral PFC is known to play a prominent role in cognitive control over memory retrieval, especially for the selection of relevant memories (Menon 2016); this could be mediated by more dorsomedial PFC regions (Hippocampus-System) that connect to the hippocampus *via* the entorhinal cortex, where object details are stored (Preston and Eichenbaum 2013).

## Methods and empirical predictions

I have argued that the Amygdala- and Hippocampus-Systems have complementary functions in self-modeling and world-modeling that are integrated during everyday cognition *via* numerous interactions along the individual expansion gradients leading to an entanglement of Amygdala-System’s hierarchical self-model and the Hippocampus-System’s world model. MPE might be a special case where the constraints of this entanglement are relaxed, making it particularly interesting for neuroscientific research, e.g. to test proposed functional consequences of the dual origin hypothesis and to identify a potential mechanism for inducing MPE modes.

Although promising computational models for MPE have been proposed (Sandved-Smith *et al*. 2021; Prest 2025; Sandved-Smith 2025), there are currently no clear neuroscientific predictions on how MPE can be reliably elicited in experimental setups. Developing such a neuroscientific, mechanistic model is not unrealistic, as similar progress has been made for equally challenging phenomena, such as out-of-body experiences (Blanke *et al*. 2004; Blanke and Metzinger 2009). Such a mechanistic model would be required because MPE appear as spontaneous and unintended events, e.g. during sleep or specific forms of advanced meditation, which might be rare and hard to induce in controlled settings where we can measure brain activity.

There are currently no reliable interventions available to consistently induce MPEs. While certain pharmacological manipulations are known to affect the sense of self perception their mechanisms of action are not well understood, e.g. even substances that target the same receptor type can lead to different experiential outcomes (e.g. different types of serotonin receptor agonists such as lysergic acid diethylamide, dimethyltryptamine, and 5-methoxy-*N*,*N*-dimethyltryptamine lead to a range of different responses from vivid hallucinations to ego dissolution). Another option would be brain stimulation. Increasing inhibition in the striatum could reduce goal-directed and habitual epistemic and instrumental actions, as suggested by preliminary data where focused ultrasound of the caudate nucleus has been used to support focus during meditation practice (Cain *et al*. 2024). An alternative target could be the anterior insula due to its role in the sense of self agency and presence (Seth *et al*. 2012). Empirical evidence suggests the insula does not create a static mapping of an interoceptive self-model; it is highly dynamic in response to interoceptive data and task demands (Gogolla *et al*. forthcoming, for review, see Flavell *et al*. 2022) and the self-model changes across the lifespan (Musculus *et al*. 2021). Direct stimulation of the dorsal insula was shown to induce states of clarity and bliss (Nencha *et al*. 2022), affecting the self-model potentially by attenuating prediction errors in the insular cortex, which would downregulate input into the central amygdala, decreasing action initiation and predictions along the exteroceptive parts of the Amygdala-System (Sladky *et al*. 2024). However, even if the correct stimulation targets are identified and reached, inducing MPE may not be reliable, as the success of brain stimulation heavily depends on the brain’s ongoing intrinsic activity (Grosshagauer *et al*. 2024).

One way to target intrinsic brain activity would be to instruct participants to employ self-regulation techniques, such as instructed meditation. Speculatively, two common forms of meditation could target complementary systems: first, focused attention meditation training has been linked to increases of interoceptive accuracy and brain activation in interoceptive brain areas, reviewed in Manuello *et al*. (2016), optimizing regions of the Amygdala-System (Fox *et al*. 2016), potentially leading to reduced anxiety and pain sensitivity (Zeidan *et al*. 2014, Zeidan *et al*. 2011). Second, open monitoring meditation (Laukkonen and Slagter 2021) could also lead to the attenuation of phenomenal self-experience (Limanowski and Friston 2020) as it reduces the identification with one’s actions, which could increase the relative influence of the Hippocampus-System. This alone is not a solution *per se*, as the Hippocampus-System overlaps with the default mode resting state network, which is involved in mind wandering and distraction (Lutz *et al*. 2008). Recollection of self-referential autobiographic memories might reinitiate Amygdala-System processes *via* self-referential episodic memories, suggesting that these two forms of meditation in isolation might not be sufficient to induce MPE. However, there is a third type of meditation practice, where the distinction between the first and third person decreases, recognized as nondual meditation by both contemplative traditions and neuroscientists (Dunne 2011; Laukkonen and Slagter 2021; Metzinger 2024). While this form of meditation could be a candidate state to functionally map MPE, there are a number of challenges: first, even advanced meditators fail to reliably reproduce nondual states, particularly inside a noisy MRI environment (Sladky *et al.*, unpublished data). Second, fMRI relies on contrasting effects (e.g. habitual phenomenal experience versus MPE) with precise temporal information on state transitions, which is typically obtained through the participant’s behavioral feedback. However, this feedback could disrupt the immersion in MPE. Third, relying on self-reports alone can lead to invalid results biased by the subjective expectations of participants and researchers.

An alternative approach would be to systematically test brain states hypothesized to play a role in MPEs by using them as training goals for real-time fMRI neurofeedback (NFB). NFB has been successfully employed as a tool for sham-controlled, double-blind interventions, not only in clinical applications but also in neuroscientific experiments (Sitaram *et al*. 2017). After the experiments, participants could report their experiential states using standardized questionnaires. Evidence supporting the involvement of a specific brain network would be provided if participants report experiences consistent with the expected MPE phenomenology while receiving a training signal aligned with the hypothesis, as opposed to a sham training signal from an unrelated brain network or another participant.

This approach addresses the three limitations mentioned earlier: first, participants do not need prior meditation experience, as they only need to follow the NFB training stimulus. Second, the known timing of the (real or sham) NFB signal allows for hypothesis-driven fMRI analyses, which provide greater statistical power compared to purely exploratory, data-driven analyses. Third, double-blinded experiments help reduce biases stemming from participants’ or researchers’ expectations.

**Figure 2:**
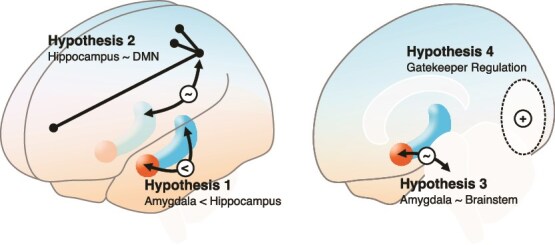
Hypothetical targets for real-time fMRI Neurofeedback to induce MPE modes. Hypothesis 1. Selective upregulation of hippocampus minus amygdala blood oxygen level dependent (BOLD) response (differential feedback).—Hypothesis 2. Reducing correlation between the hippocampus and the default mode network (functional connectivity feedback).—Hypothesis 3. Increasing/decreasing correlation between core Amygdala-/Hippocampus-System areas (anterior insula, ACC, etc.) and the brainstem.—Hypothesis 4. Increasing local correlation of the visual cortex or other potential gatekeeper regions.

Conditional on our theoretical analysis I suggest four potential NFB sources that could function as a suitable training signal for MPE (Fig. 2).

-
  Hypothesis 1. Differential feedback of the hippocampus > amygdala contrast tests the hypothesis that MPE results from relatively lower Amygdala-/higher Hippocampus-System core activation. The feasibility of amygdala and hippocampus-based NFB training in naïve participants has been demonstrated (Scharnowski *et al*. 2015; Watve *et al*. 2024). This aspect focusses on nonself-related experiences during MPE (Metzinger 2024). Consistent with this hypothesis, empirical results could suggest to include (a) additional Amygdala-System (e.g. insula) and Hippocampus-System regions (e.g. entorhinal cortex) to increase the temporal signal-to-noise ratio or (b) focusing on specific amygdalar subnuclei/hippocampal subfields taking into account functional differentiation gradients (Borne *et al*. 2023) to increase specificity.
-
  Hypothesis 2. If downregulation of self-relevant Amygdala-System regions proves insufficient, an alternative approach could involve reducing input to the hippocampus from DMN regions. During MPE, there should be reduced involvement of Hippocampus-System’s lower hierarchical layers, such as the DMN, which could otherwise provide autobiographical details that reactivate the self-model. Such peripheral Hippocampus-System deactivations would align with the observed strong electroencephalography (EEG) alpha suppression over occipital and parietal brain areas in meditators who experienced a cessation of conscious experience during meditation (Chowdhury *et al*. 2023). Without perturbations from autobiographic, episodic memory hippocampal processes could be focused on more abstract, generalizable, less ego-centric forms of content. Phenomenologically, this aspect might particularly address the experience of endless space and knowledge as such during MPE, described by some participants (Metzinger 2024).
-
  Hypotheses 3. In an early proposal on MPE, Thomas Metzinger suggested that the experiential content of pure awareness is a predictive model of one’s tonic alertness as regulated by the ascending reticular activating system in the brainstem (Metzinger 2020), which includes several nuclei that are required for the synthesis and release of key neurotransmitters. Correlation between these brainstem nuclei and Amygdala-System (e.g. anterior insula) or Hippocampus-System regions (e.g. parahippocampus) could be a marker for how phasic and tonic arousal entrain whole-brain states and experiences—and thus could be a viable training target. More precisely, Hippocampus-System’s dorsal parahippocampus is assumed to be regulated by phasic arousal, mediated by lemnothalamic pathways from the reticular activating system of the lower brainstem and consolidated during rapid eye movement (REM) sleep. Amygdala-System’s core areas, on the other hand, are regulated by collothalamic pathways from the midbrain and consolidated during non-REM sleep (Tucker *et al*. 2025).
-
  Hypothesis 4. Failure of other hypotheses might suggest that directly regulating the core Amygdala-/Hippocampus-System balance or input is either insufficient or unfeasible for the participant. However, this does not disprove the overall approach, as a more relevant and stable training signal could come from auxiliary brain regions that help stabilize the neural dynamics necessary for MPE. For instance, training with a striatal NFB signal could enhance the participant’s focus on internal processes. Another viable option is using fMRI NFB to self-regulate the dopaminergic midbrain (Kirschner *et al*. 2018). Alternatively, intrinsically regulating visual cortex activation (Scharnowski *et al*. 2014), e.g. increasing the correlation across all voxels, could be related to the experiential reports of radiance and clear light during MPE (Metzinger 2024). Successful regulation of these gatekeeper regions could increase the opaqueness of the other MPE phenomena.

## Conclusion

If the Amygdala-System (plus striatum, brainstem, and some other subcortical nuclei) is the ‘hidden spring’ of egocentric phenomenal experiences, centered around drives, needs, and motivation—then the Hippocampus-System could be an ‘endless ocean’ of allocentric phenomenal experiences. While there is flexibility of the self-model embodied in the Amygdala-System, it is always constrained by physiological needs, limiting its degrees of freedom. The Hippocampus-System, on the other hand, would be less constrained; its architecture resembles an ideal system for unconstrained associative learning, which has also been proposed as another evolutionary driver for consciousness (Jablonka and Ginsburg 2022).

I have proposed using real-time fMRI NFB to upregulate core Hippocampus-System areas, encouraging brain processes that optimize knowledge representation at the most abstract level, when free from the distractions of self-regulation and bodily experiences. Empirical evidence supports the idea that core Hippocampus-System areas encode sensory data features and their relationships in a highly abstract and compressed manner. For example, abstract predictive models are created in the hippocampus (Stachenfeld *et al.* 2017) and the entorhinal cortex (Ouchi and Fujisawa 2024) and it has been shown that the hippocampus generalizes by reducing the complexity of data, leveraging its flexible and abstract model architecture (Courellis *et al.* 2024). The phenomenal experience of this neurocomputational process is speculative, but it might involve a nonegocentric, nonagentic sense of open-endedness and positive valence. This subtle experience is often overlooked or remains transparent, overshadowed by more prominent self-related experiences, when viewed through the lens of the ego-centric self-model. When unconstrained by a self-model (due to attenuated interoception) or the need for action (due to attenuated exteroception and agency), the brain’s only option may be to focus on optimizing its internal world model. This could give rise to the simplest form of wakeful phenomenal experience.

## Data Availability

This submission is a theoretical analysis and does not contain new empirical data.

## Footnotes

1
When it comes to interoception, Mark Solms notes that the insula is not necessary for the experience of feeling the body. “Patient B., whose insular cortices were entirely destroyed, experienced body feelings as well as emotional feelings. He reported feeling pain, pleasure, itch, tickle, happiness, sadness, apprehension, irritation, caring and compassion, and he behaved in ways consonant with such feelings when he reported experiencing them. He also reported feelings of hunger, thirst, and desire to void, and behaved accordingly. He yearned for play opportunities, for example, playing checkers, visiting with others, going for walks, and registered obvious pleasure when engaged in such activities as well as disappointment or even irritation when the opportunities were denied. [...] Given the impoverishment of his imagination, Patient B.’s existence was a virtually continuous ‘affective’ reaction to his own body states and to the modest demands posed by the world around him, undampened by high-order cognitive controls [...].” (Solms 2021)
